# Nasal decongestant and chronic headache: a case of naphazoline overuse headache?

**DOI:** 10.12688/f1000research.2-237.v1

**Published:** 2013-11-11

**Authors:** Cherubino Di Lorenzo, Gianluca Coppola, Valeria La Salvia, Francesco Pierelli

**Affiliations:** 1 Don Carlo Gnocchi Onlus Foundation, Milan, 20100, Italy; 2 Department of Neurophysiology of Vision and Neurophthalmology, G.B. Bietti Foundation IRCCS, Rome, 00100, Italy; 3 Department of Medical and Surgical Sciences and Biotechnologies and ICOT, “Sapienza” University of Rome Polo Pontino, Latina, 04100, Italy; 4 IRCCS-Neuromed, Pozzilli (IS), 86077, Italy

## Abstract

**Background:** Chronic headache is an incapacitating condition afflicting patients at least for 15 days per month. In the most cases it is developed as a consequence of an excessive use of symptomatic drugs.

**Case: **Here we report the case of a 34 year-old man suffering from chronic headache possibly related to the overuse of naphazoline nitrate nasal decongestant, used to treat a supposed chronic sinusitis. However, the patient did not suffer from sinusitis, but from a medication overuse headache (ICHD-II 8.3; ICD-10 44.41) that appeared to be due to excessive use of naphazoline.

**Conclusion: **The use of naphazoline nitrate may result in an analgesic effect upon first use, through activation of adrenergic and opioidergic systems, followed by a pro-migraine effect via a late induction of an inflammatory cascade, modulated by nitric oxide and arachidonic acid. The observation that naphazoline detoxification relieved the patient’s headache, indicates that prolonged use of naphazoline may cause chronic headaches. Therefore, physicians should ask for details on the use of nasal decongestants in patients complaining of chronic headache, as they could potentially be suffering from a medication-overuse headache.

## Introduction

Chronic headache (≥ 15 days/month) is an incapacitating condition affecting the 4–5% of the general population ^1^, and can be primary (in which case the headache is not due to other causes but is the disease per se) or secondary (i.e., the symptom of another condition) ^2^. In 80% of cases, the chronic headache is a secondary effect caused by an excessive intake of drugs, mainly analgesics ^3^. The excessive intake of pain-killers for more than consecutive 3 months can cause medication overuse headache (MOH), a secondary form of chronic headache, widespread among migraineurs ^2^.

Here we report the case of a patient suffering from a chronic headache related to the overuse of naphazoline nitrate, an over the counter nasal decongestant that the patient spontaneously used to treat a self diagnosed chronic sinusitis.

## Case report

A 34 year-old man came to our Outpatient Headache Clinic in Latina ICOT hospital, (Italy) presenting a daily orbito-frontal bilateral headache, that he had been suffering from since he was 18. The patient had assumed it was a chronic sinusitis.

The patient had not recently undergone any diagnostic evaluation. The only examination that had been performed for his condition was a brain MRI, performed 8 years ago in a private clinic. This MRI detected no abnormalities, except a soft radio-opacity of the paranasal and zygomatic sinuses, and hypertrophy of the nasal mucosa. The general practitioner (GP) did not advice any specific treatment after this MRI-based diagnosis, and patient continued to use naphazoline.

The headache experienced by the patient was bilateral, throbbing, sometimes very severe, and associated with nausea, vomiting, photophobia, osmophobia and worsened by head movements.

The patient had been treated by his GP with medication to relieve the headache (metamizole, rizatriptan, zolmitriptan, acetylsalicylic acid, nimesulide, ibuprofen, naproxen sodium), consumed more than once a day, until the age of 24. Since then, he had stopped the consumption of such painkillers because he found that naphazoline nitrate nasal spray was more effective. He began with 2 shots (0,14 mg of drug for any shot) for each nostril 3 times a day, and at the time of presentation, due to pain recurrence, was using the spray 5–6 times a day, and experiencing immediate, yet temporary, relief of the symptoms every time. When asked, the patient said that he had used naphazoline nitrate nasal spray on an occasional basis since the age of 16, in order to self-medicate for self-diagnosed chronic rhinitis.

During the patient’s visit to the clinic, respiratory examination, blood pressure, heart rate, mental status, reflexes, sensory system, cranial nerve, motor system, gait and coordination were normal. Since his headache characteristics were suggestive for a migraine-like headache, further evaluations were required to exclude the diagnosis of an acute sinusitis. Through a CT scan, a relapse of chronic sinusitis was excluded. The patient was then referred to an otolaryngologist, who suggested a detoxification from naphazoline nitrate through the use of aerosol therapy, with mucolytics and steroids, and thermal water spray.

The chronic headache disappeared following the treatment suggested by otolaryngologist. After three months of headache diary recording and clinical re-evaluation, the clinical picture was dramatically changed: patient presented only sporadic attacks of migraine without aura (ICHD-II 1.1). By an anamnesis re-evaluation it emerged that an episodic headache arose in childhood and worsen over the years, until it became chronic by the age of 18. One year after naphazoline detoxification, the patient has suffered from a few attacks during the year, treated with triptans.

## Discussion

The case we report is suggestive for a chronic headache secondary to naphazoline nitrate overuse, since drug discontinuation interrupted the clinical symptoms. It is not clear however, whether the development of the chronic headache was due to a well-known naphazoline nitrate adverse event (AE) of inducing headache ^4^, or whether it was due to a MOH-like occurance, since the patient consumed this drug to treat his headache. Indeed, patient experienced a relief on his headache by naphazoline, this was the reason he supposed, erroneously, to suffer of chronic sinusitis. The anti-migraine effect of naphazoline on our patient raises a question: is it right to regard naphazoline as a migraine medication?

Naphazoline is a sympathomimetic drug, an imidazolinic derivate with marked alpha-adrenergic activity ^4^. It enhances the release of noradrenaline from adrenergic termination, immediately relieving the nasal congestion thanks to its vasoconstricting action on the vessels of nasal mucosa ^5^. Because of its adrenergic activity, this drug can also produce adverse effects, like rhinitis medicamentosa, hypertension, headache and acute depression of central nervous system with marked sedation ^4^. Moreover, cases of ischemic and hemorrhagic stroke secondary to naphazoline have also been reported, mediated by the alpha1 and alpha2 adrenergic vasoconstrictive effect that is also exerted on brain vessels ^6, 7^.

**Figure 1.**
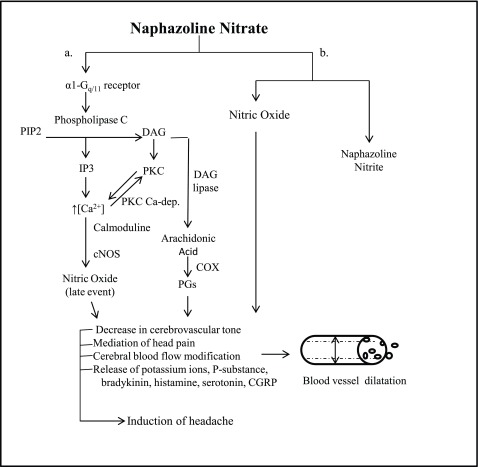
Late events induced by naphazoline that could cause headache. **a**. Naphazoline activates α1 receptors that are associated with G-proteins that cause the activation of phospholipase C. Phospholipase C cleavesphosphatidyl-inositol 4,5-bisphosphate (PIP2) into two second messengers, inositol 1,4,5-trisphosphate (IP3) and diacylglycerol (DAG) which in turn cause an increase in the level of calcium and protein kinase C. Diacylglycerol, by DAG lipase, takes part in the creation of arachidonic acid which is a precursor in the production of prostaglandins (PGs), mediated by cyclooxygenase (COX). The higher concentration of intracellular calcium allows an increase in the complex of calcium-calmodulin and therefore the activation of constitutive nitrous oxide synthetase (cNOS) with the generation of nitric oxide ^8, 9^. **b**. Naphazoline nitrate can contribute to the production and release of further NO by a chemical reduction of the naphazoline salt. Prostaglandins and nitric oxide contribute to the activation of nociceptors and the transmission of the pain pulse from the periphery to the centre ^10^. Consequently there is a release of substances such as potassium ions, P-substance, bradykinin, histamine, serotonin and CGRP that keep nociceptors active and result in vasodilatation and extravasation of plasma proteins from the vessels.

Naphazoline can trigger headaches because of its adrenergic activity ( Figure 1). Alpha1 receptors are associated with G-proteins that generate a cascade of events leading to the production of arachidonic acid (AA) and nitric oxide (NO). AA and NO release leads to a late inflammatory vasodilatation ^8, 9^ that could induce a migraine attack ^10^. Moreover, it is also possible that the same naphazoline nitrate, consumed by our patient, could have contributed to the production and release of further NO by a chemical reduction of the naphazoline salt. In fact, NO donors are currently used to induce migraine attacks in clinical and experimental settings ^11^.

**Figure 2.**
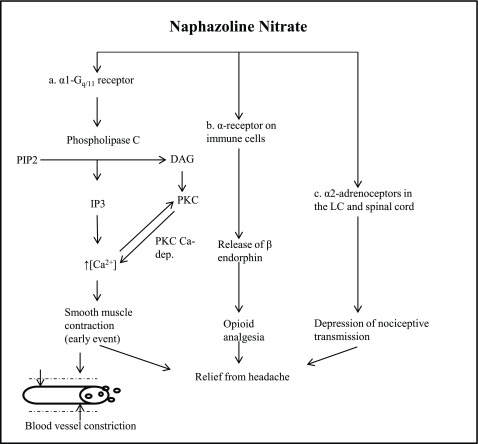
Possible early events that induce peripheral analgesic effect. **a**. Naphazoline activates alpha1 receptors that are associated with G-proteins that cause the activation of phospholipase C. Phospholipase C cleaves phosphatidyl-inositol 4,5-bisphosphate (PIP2) into inositol 1,4,5-trisphosphate (IP3). Consequently there is a smooth muscle contraction due to an increase of intracellular calcium. **b**. Alpha-Receptors on immune cells release β-endorphins that cause opioid analgesia. **c**. Activation of alpha2 adrenoceptors in the Locus Coeruleus and spinal cord provokes the depression of nociceptive transmission ^12^.

However, despite the late effect as a migraine trigger factor, naphazoline might also have an early action as an anti-migraine agent acting on alpha receptors of muscle, immune cells, Locus Coeruleus and spinal cord ( Figure 2). In fact, alpha2 adrenergic receptors have a peripheral analgesic effect, thanks to the activation of opioidergic receptors, via endogenous opioid release ^12^. Moreover, both alpha1 and alpha2 adrenergic receptors agonists have been proposed to be specifically useful for the treatment of migraine by mechanisms that mediate the early vasoconstrictive effect related to their activation ^8^, similarly to triptans that carry out an analgesic action through the serotoninergic agonism that also mediates vasoconstriction.

Taking together these two opposite effects, it is possible to speculate that naphazoline can have a very early analgesic effect, due to the initial adrenergic activation, and a late migraine-inducing effect, that promotes the recurrence of headache by NO and arachidonic acid modulation.

We could therefore presume that in our patient, in a similar manner to the "triptans effect", naphazoline could exert an antimigraine action but also induce a rebound chronic headache due to medication overuse and/or a proinflammatory cytokine-mediated headache induction. Indeed, our patient had experienced such a sudden analgesic effect; otherwise he would not have continued to take naphazoline.

The observation that naphazoline detoxification leads to the interruption of headache is in line with the guidelines of the International Headache Society that prescribes treatment discontinuation for drugs that can directly induce headache and in cases of MOH ^3^.

In conclusion, although naphazoline may cause headache as an AE, it may also have an early analgesic effect in migraine, as experienced by our patient. Our observation enlarges the spectrum of the drugs that can cause MOH and suggests that physicians should pay attention to the consumption of nasal decongestants in their chronic headache patients.

## Consent

Written informed consent for publication of this case report was obtained from the patient.
