# Supplementary material for: Nasal decongestant and chronic headache: a case of naphazoline overuse headache?
Source: F1000Res. 2013 Nov 11;2:237. [Version 1] doi: 10.12688/f1000research.2-237.v1 (PMC4111119; doi:10.12688/f1000research.2-237.v1)
Supplement: Peer review history

# Peer review: Referee response for version 1

**DOI:** 10.5256/f1000research.2342.r4464

**Published:** 2014-04-23

**Authors:** Mark Obermann

**Affiliations:** 1 Department of Neurology, University of Duisburg-Essen, Duisburg-Essen, Germany

The authors present an interesting case report of chronic headache possibly related to the nasal decongestant naphazoline. They clearly and reasonably argue that naphazoline may have caused headache as a well known side effect of this drug and/or due to a similar mechanism known from other pain medications (analgesics/triptans/opioids) in terms of medication overuse headache. They laboriously explain the possible pharmacological mechanisms of action that may have caused headache over the long run, as well as early pain relief mechanisms that led to the continued intake of this drug in the first place. All this is highly hypothetical as correctly pointed out by the authors and may or may not stress the possibility of many different drugs that may cause medication overuse headache or may contribute to the development of chronic headache in particular patients.

This case report provides a nice impulse for further research in this direction and emphasizes the possibility of other medications that may cause medication overuse headache beyond triptans, analgesics, etc. This case should make us clinicians more alert for these matters in order to better treat our patients with chronic headache.

Many pre-clinical and clinical studies will be needed before any of the assumptions made by the authors can be confirmed or discarded.

I have read this submission. I believe that I have an appropriate level of expertise to confirm that it is of an acceptable scientific standard.

# Peer review: Referee response for version 1

**DOI:** 10.5256/f1000research.2342.r2801

**Published:** 2013-12-17

**Authors:** Dimos Mitsikostas

**Affiliations:** 1 Neurology Department, Athens Naval Hospital, Athens, Greece

This is an interesting report showing that naphazoline use was associated with chronic headache in one patient. The patient deserved further evaluation to exclude other brain pathologies related to chronic headache (e.g. no brain MRI or MRA was performed). The authors made a long and nice hypothesis regarding the potential pharmacological induction of headache which, however, needs experimental documentation.

I have read this submission. I believe that I have an appropriate level of expertise to confirm that it is of an acceptable scientific standard, however I have significant reservations, as outlined above.

# Response

**Published:** 2013-12-17

**Authors:** Cherubino Di Lorenzo

**Affiliations:** Department of Neuroscience, Palliative Home-Care Unit for Brain Tumor Patients, Don Gnocchi Foundation, Milan, Italy

We thank Dr Mitsikostas for his kind response.

The decision to not repeat the MRI (the first, performed eight years before, was negative although the clinical picture was already arisen) was a consequence of the very positive outcome experienced by patient after discontinuation of nasal spray. In fact, both the waiting list for CT scan and otolaryngologist consultation were very little, if compared to the MRI list, thus the ‘ *ex adiuvantibus*’ confirmation of our suspect induced patient to not require another magnetic resonance because he preferred to avoid the discomfort related to the procedure.

We agree that our speculations have to be sustained by further experimental confirmations. However, it is interesting to highlight that the information pamphlet of several nasal decongestants reported headache as a common side effect, but we do not find any notice of it in literature. On the other side, never it was suggested a sudden positive effect for naphazoline on migraine headache. We hope that our report will be the cue for further in-depth analysis on the topic.

# Peer review: Referee response for version 1

**DOI:** 10.5256/f1000research.2342.r2380

**Published:** 2013-12-12

**Authors:** Christian Wöber

**Affiliations:** 1 Department of Neurology, Medical University of Vienna, Vienna, Austria

An interesting case report which should be confirmed by further studies.

I have read this submission. I believe that I have an appropriate level of expertise to confirm that it is of an acceptable scientific standard.
